# Supplementary material for: Identification of druggable binding sites and small molecules as modulators of TMC1
Source: Commun Biol. 2025 May 13;8:742. doi: 10.1038/s42003-025-07943-x (PMC12075566; doi:10.1038/s42003-025-07943-x)
Supplement: Supplementary file 3 — Description of Additional Supplementary Files [file 42003_2025_7943_MOESM3_ESM.docx]

Description of Additional Supplementary Files

**File Name:** Supplementary Data

**Description:** The source data and statistical analysis behind Fig 8B, Fig S2, Fig S4, Fig S7 and Fig S8.
