# Supplementary material for: Identification of druggable binding sites and small molecules as modulators of TMC1
Source: Commun Biol. 2025 May 13;8:742. doi: 10.1038/s42003-025-07943-x (PMC12075566; doi:10.1038/s42003-025-07943-x)
Supplement: Supplementary file 4 — Reporting summary [file 42003_2025_7943_MOESM4_ESM.pdf]

nature portfolio

Corresponding author(s): Artur Indzhykulian
Pedro De-la-Torre
David Ramirez

Last updated by author(s): 2025/08/03

Reporting Summary

Nature Portfolio wishes to improve the reproducibility of the work that we publish. This form provides structure for consistency and transparency in reporting. For further information on Nature Portfolio policies, see our Editorial Policies and the Editorial Policy Checklist.

Statistics

For all statistical analyses, confirm that the following items are present in the figure legend, table legend, main text, or Methods section.

n/a	Confirmed	
☐	☒	The exact sample size (*n*) for each experimental group/condition, given as a discrete number and unit of measurement
☐	☒	A statement on whether measurements were taken from distinct samples or whether the same sample was measured repeatedly
☐	☒	The statistical test(s) used AND whether they are one- or two-sided<br>*Only common tests should be described solely by name; describe more complex techniques in the Methods section.*
☒	☐	A description of all covariates tested
☐	☒	A description of any assumptions or corrections, such as tests of normality and adjustment for multiple comparisons
☐	☒	A full description of the statistical parameters including central tendency (e.g. means) or other basic estimates (e.g. regression coefficient) AND variation (e.g. standard deviation) or associated estimates of uncertainty (e.g. confidence intervals)
☐	☒	For null hypothesis testing, the test statistic (e.g. *F*, *t*, *r*) with confidence intervals, effect sizes, degrees of freedom and *P* value noted<br>*Give P values as exact values whenever suitable.*
☒	☐	For Bayesian analysis, information on the choice of priors and Markov chain Monte Carlo settings
☒	☐	For hierarchical and complex designs, identification of the appropriate level for tests and full reporting of outcomes
☒	☐	Estimates of effect sizes (e.g. Cohen's *d*, Pearson's *r*), indicating how they were calculated

Our web collection on statistics for biologists contains articles on many of the points above.

Software and code

Policy information about availability of computer code

Data collection	Confocal data was collected in Leica LASX. Structural analysis, docking, and molecular dynamics simulations were carried out using the Liprep, Glide, Epik, Phase and KNIME suites of the Software Maestro (Schrodinger). The OPLS3 and S-OPLS force fields were used for molecular dynamics simulations.
Data analysis	Image analysis was carried out in ImageJ and Cellpose. Statistical analysis and data representation was carried out in Excel, and GraphPad Prism. Domain structure prediction, and AlphaFold2 simulations were carried out using the ColabFold v1.5.2-patch server. The binding energies were analyzed with the Maestro software and the KNIME software. 3 replicates were carried out for 25 ns molecular dynamics simulations.

For manuscripts utilizing custom algorithms or software that are central to the research but not yet described in published literature, software must be made available to editors and reviewers. We strongly encourage code deposition in a community repository (e.g. GitHub). See the Nature Portfolio guidelines for submitting code & software for further information.

Data

Policy information about availability of data

All manuscripts must include a data availability statement. This statement should provide the following information, where applicable:

- Accession codes, unique identifiers, or web links for publicly available datasets
- A description of any restrictions on data availability
- For clinical datasets or third party data, please ensure that the statement adheres to our policy

All data supporting the findings of this study are available within the paper and its Supplementary Information. The PDB file containing the coordinates of the simulated MET channel complex, 25 ns simulation replicates, and the 3D-pharmacophores used in this study are available for download from the Ramirez Lab Github repository: https://github.com/ramirezlab/Drug-design-targeting-TMC1.

Research involving human participants, their data, or biological material

Policy information about studies with human participants or human data. See also policy information about sex, gender (identity/presentation), and sexual orientation and race, ethnicity and racism.

Reporting on sex and gender	Not Applicable.
Reporting on race, ethnicity, or other socially relevant groupings	Not Applicable.
Population characteristics	Not Applicable.
Recruitment	Not Applicable.
Ethics oversight	Not Applicable.

Note that full information on the approval of the study protocol must also be provided in the manuscript.

Field-specific reporting

Please select the one below that is the best fit for your research. If you are not sure, read the appropriate sections before making your selection.

☒ Life sciences ☐ Behavioural & social sciences ☐ Ecological, evolutionary & environmental sciences

For a reference copy of the document with all sections, see nature.com/documents/nr-reporting-summary-flat.pdf

Life sciences study design

All studies must disclose on these points even when the disclosure is negative.

Sample size	Sample sizes were based on similar studies from other groups.
Data exclusions	No Data was excluded from analysis.
Replication	All docking poses were analyzed for a maximum of 10 different posses. 3 replicates were carried out for 25 ns molecular dynamics simulations. All compounds were evaluated with 6 different experiments, with a maximum of 4 biological replicates. Some compounds were evaluated one time only.
Randomization	Animals were randomly allocated to experimental groups in this study.
Blinding	For the FM1-43 uptake testing, the experimenter was unaware of the biophysical properties of the selected compunds by in silico analysis

Reporting for specific materials, systems and methods

We require information from authors about some types of materials, experimental systems and methods used in many studies. Here, indicate whether each material, system or method listed is relevant to your study. If you are not sure if a list item applies to your research, read the appropriate section before selecting a response.

Materials & experimental systems

n/a	Involved in the study
☒	☐ Antibodies
☒	☐ Eukaryotic cell lines
☒	☐ Palaeontology and archaeology
☐	☒ Animals and other organisms
☒	☐ Clinical data
☒	☐ Dual use research of concern
☒	☐ Plants

Methods

n/a	Involved in the study
☒	☐ ChIP-seq
☒	☐ Flow cytometry
☒	☐ MRI-based neuroimaging

Animals and other research organisms

Policy information about studies involving animals; ARRIVE guidelines recommended for reporting animal research, and Sex and Gender in Research

Laboratory animals	Wild-type CD1 mice from both sexes were used in the study. All animals were in-house bred.
Wild animals	The study did not involve wild animals
Reporting on sex	Experimental animals of both sexes were used at random. Sex based analysis was not carried out.
Field-collected samples	Samples were not collected from the field.
Ethics oversight	All procedures were conducted in compliance with ethical regulations approved by the Institutional Animal Care and Use Committee of Mass Eye and Ear.

Note that full information on the approval of the study protocol must also be provided in the manuscript.

Plants

Seed stocks	Not Applicable.
Novel plant genotypes	Not Applicable.
Authentication	Not Applicable.
